# Pleiotropic effects of red and purple pericarp genes on seed coating patterns, flavonoids, dormancy, and germination in rice

**DOI:** 10.1093/g3journal/jkaf158

**Published:** 2025-07-18

**Authors:** Wenwu Tang, Min Guo, Yue Zhu, Rupak Chakraborty, Bhupinder S Batth, Kamal Bhattarai, Guiquan Zhang, De-Yu Xie, Xing-You Gu

**Affiliations:** Agrinomy, Horticulture & Plant Science Department, South Dakota State University, Brookings, SD 57007, United States; Agrinomy, Horticulture & Plant Science Department, South Dakota State University, Brookings, SD 57007, United States; Department of Plant and Microbial Biology, North Carolina State University, NC 27695, United States; Agrinomy, Horticulture & Plant Science Department, South Dakota State University, Brookings, SD 57007, United States; Agrinomy, Horticulture & Plant Science Department, South Dakota State University, Brookings, SD 57007, United States; Agrinomy, Horticulture & Plant Science Department, South Dakota State University, Brookings, SD 57007, United States; Agricultural College, South China Agricultural University, Guangzhou 510642, China; Department of Plant and Microbial Biology, North Carolina State University, NC 27695, United States; Agrinomy, Horticulture & Plant Science Department, South Dakota State University, Brookings, SD 57007, United States

**Keywords:** pleiotropy, flavonoid, seed dormancy, germination, rice

## Abstract

Seeds are coated with pigments presumably to promote plant adaptation. To understand the adaptive mechanisms of seed pigment traits, allelic variants of the red (*Rc/rc*) and purple (*Pb/pb*) pericarp color genes were assembled into the same genetic background to identify the trait development patterns and pleiotropies of the loci on seed flavonoids, dormancy, and germination in rice (*Oryza sativa*). Nonallelic recombination and epistasis of the loci dictated 4 patterns of the trait development from 5 to 40 d post-anthesis. The *Rc*- and *Pb*-controlled pigments were synthesized in the same lower epidermal cells but compartmented in the cells and lignified wall area, respectively, after 10 d. Four flavan-3-ols (catechin, epicatechin, and their dimeric procyanidins) and anthocyanins (AC) were detected in the *Pb* and *Rc* systems, respectively, with catechin being most abundant. Both genes affected seed primary dormancy, and imbibition and germination velocities of the dormancy-released seeds. Additive effects of the loci contributed most to the variances for all the pleiotropic traits, the development time and its interaction with the additive components influenced the flavonoid contents, and the additive-by-additive epistasis modified the AC content and dormancy level. Thus, seed pigment traits influence plant adaptation likely through a series of pleiotropies, including the coat structure, enhanced dormancy, and reduced germination speed. The differences between the *Rc* and *Pb* loci in the flavonoid type/content and the size of pleiotropic effects could partly explain the predominance of red pericarp-colored genotypes in wild and weedy rice and in pigmented specialty cultivars.

## Introduction

Seeds vary in the coat (testa, pericarp, or hull) color with plant genotypes. Adaptive significance of the seed pigment traits was related to underlying chemicals, coat structures, or association with seed dormancy. The chemicals are flavonoids acting as antioxidants to protect seeds from predation by herbivores or damage by ultraviolet irradiation and microbes (Shirley 1998; Lepiniec et al. 2006). The pigments in the testa may alter the coat biophysical properties, water and gas permeabilities, and germination speed in mustard (*Brassica* spp.) and legume (Fabaceae family) plants (Debeaujon et al. 2000; Smýkal et al. 2014; Demonsais et al. 2020). The red pericarp-colored genotypes have stronger seed dormancy than the white ones in wheat (*Triticum aestivum*; Nilsson-Ehle 1914), millet (*Panicum miliaceum*; Khan et al. 1997), and weedy rice (*Oryza sativa*; Gu et al. 2003). Many species evolved with 2 or more seed pigment traits (e.g. red and purple pericarp and black hull colors in rice), each controlled by 1 or a few genes. It is unclear if different seed pigment traits vary in contribution to the plant adaptation or if seed pigmentation genes differ in pleiotropic effect on the associated biophysical and physiological traits. This research used the red and purple pericarp color traits in rice as a model system to address the questions.

A pericarp (fruit coat) develops from an ovary wall after fertilization and replaces the testa (seed coat) to protect the seed during its development, dispersal, storage in soil seed banks, and germination. There are red and purple pigment systems expressed in the pericarp tissue of rice. The red pigments are condensed tannins (oxidized proanthocyanins or PA), and red-pigmented genotypes distributed in wild (*Oryza rufipogan*) and weedy (*O. sativa*) rice, and specialty cultivars (*O. sativa* and *O. glaberrima*; Oka 1988). Genetic analysis identified the *Rc/rc* and *Rd/rd* alleles responsible for the phenotypic variation from red (*Rc_Rd*_), brown (*Rc_rdrd*) to white (*rcrcrdrd*) colors (Hsieh and Chang 1964). *Rc* is a regulatory gene encoding a basic helix-loop-helix (bHLH) family transcription factor (TF), while *Rd* encoded DIHYDROFLAVONOL-4-REDUCTASE (DFR) for PA synthesis (Sweeney et al. 2006; Furukawa et al. 2007; Supplementary Table 1).

The purple pigments are anthocyanins (AC). The purple-pigmented genotypes are present in some tropical landraces and specialty varieties (Mbanjo et al. 2019; Fongfon et al. 2021). Genetic analysis also identified allelic variants at 2 loci (*Pp* and *Pb*) responsible for the variation from purple (*Pb_Pp_*), brown (*Pb_pppp*) to white (*pbpbpppp*) colors (Hsieh and Chang 1964; Rahman et al. 2013). Alternative names for the *Pb* and *Pp* loci were used in different research after the 1960s (Supplementary Table 1). Two complementary DNAs (cDNAs), designated *OsB1* and *OsB2*, were isolated from a purple-colored genotype and both activated for AC synthesis in aleurone cells of rice (Sakamoto et al. 2001). Fine-mapping with a large segregating population delimited *OsB1* and *OsB2* to the *Pb* locus, a genomic region containing the Os04g47080 (*OsB1*) and Os04g47059 (*OsB2*) genes (Wang and Shu 2007), both encoding a subfamily of bHLH TFs, based on the reference (Nipponbare) genome sequence (Kawahara et al. 2013). The *Pb* complex was later mapped as *Kala4* (Maeda et al. 2014). The Os04g47059 locus is >24 kilo base pairs (kb) in genomic DNA sequence, but its cDNA is <1 kb; over-expression of the short cDNA from a nonpigmented cultivar changed the recipient's pericarp color from white to purple, supporting that *OsB2* is an underlying gene of *Pb* or *Kala4* (Oikawa et al. 2015). The *Pp* gene was named *Kala1*, which is identical to *Rd* (Maeda et al. 2014). Thus, these and other data provided evidence that *Rc* and *Pb* regulate shared pathways for the PA or AC biosynthesis, likely through the conserved MYB-bHLH-WD40 protein complex in rice (Xia et al. 2021).

The red and purple pigment systems appeared different in response to natural and domestication selections. The *Rc* alleles originated from wild rice and are spread in tropical to temperate ecotypes of weedy rice (Oka 1988; Delouche et al. 2007; Gross et al. 2010). In contrast, the *Pb* allele may originate as a mutant in a tropical ecotype (Oikawa et al. 2015). In a collection of 696 pigmented accessions of traditional varieties from Philippines, 81% are red- and 12% are purple-colored (Mbanjo et al. 2019). Thus, the pericarp colors alone cannot explain why the red-pigmented genotypes are more frequent than the purple-pigmented ones in wild and weedy rice and in the collection of traditional specialty varieties.

Pleiotropy was reported for the red pericarp genes in wheat (*R*) and rice (*Rc*). In the hexaploidy wheat, 3 *R* loci were all collocated with quantitative trait loci (QTL) for seed dormancy (SD), each accounted for 3% to 10% of the phenotypic variance (Groos et al. 2002). The *R* homoeologs encode Myb-family TFs (Himi and Noda 2005; Lang et al. 2021). In weedy rice, 2 collocated QTL accounted for 9% to 11% of the variances for SD (*qSD7-1*) or 43% to 73% of the variances for pericarp colors (*qPC7*; Gu et al. 2005). Fine-mapping delimited the *qSD7-1/qPC7* cluster to the *Rc* locus using intragenic recombinants, and the TF gene activated the flavonoid biosynthesis in the pericarp lower epidermal cell layer and promoted accumulation of abscisic acid (ABA, a dormancy-inducing hormone) in developing seeds (Gu et al. 2011). *qSD7-1* was associated with coat-imposed dormancy (Gu et al. 2015), soil seedbank longevity (Pipatpongpinyo et al. 2020) and seed aging tolerance (Prasad et al. 2023). Pleiotropy on the seed adaptive traits was not reported for the other pigmentation genes, such as *Pb* in rice and *Ant1* and *Ant2* in barley (*Hordeum vulgare*; Gordeeva et al. 2019).

Seed dormancy and germination are 2 related but distinct physiological processes and both are key early life-history traits of adaptive significance (Bewley 1997; Saatkamp et al. 2019). In addition, seed-pigmented specialty crops are being developed for potential health benefits of the secondary metabolites. In this research, the *Rc/rc* and *Pb/pb* allelic variants were assembled into the same genetic background as a set of introgression lines (ILs) to assess or quantify their effects on seed coating, biochemical and physiological properties to infer differences in contribution to plant adaptation or health benefit between the red and purple pigment traits. Specific questions addressed include: (i) developmental patterns of the red and purple pericarp color traits at morphological and histological levels; (ii) types and contents of flavonoid chemicals regulated by *Rc*, *Pb*, or both in developing and mature caryopses; (iii) main and epistatic effects of the 2 loci on primary seed dormancy in the ILs and a hybrid (F_2_) population; and (iv) the effects on germination and imbibition velocities after the dormancy is released. Results from the research were discussed regarding the predominance of red over the purple pericarp-colored genotypes in non-domesticated and domesticated pigmented rice.

## Materials and methods

### Plant genotypes, cultivation, and seed harvesting

Four isogenic lines (ILs) homozygous for the *Rc/rc* and *Pb/pb* alleles, designed IL_Rcpb_, IL_rcPb_, IL_RcPb_, and IL_rcpb_, were selected from a library of single chromosome segment substitution lines. The library was developed by backcrossing and genomic selections to introduce the segments from various donors into the background of ‘Huajinxian 74’ (*rcrcpbpb* or IL_rcpb_), an *indica*-type modern cultivar of white pericarp color (Zhang 2021). The *Rc* allele was donated by ‘BG367’, a red pericarp-colored landrace from Bangladesh, while the *Pb* allele was introduced from ‘Lianjian 33’, a purple pericarp-colored landrace from Guangdong, China. The 4 lines were grown in a greenhouse, and seeds harvested from single plants used for the following experiments. A cross between IL_rcpb_ and IL_RcPb_ was made to develop an F_2_ population to evaluate component effects of the *Rc* and *Pb* loci on SD.

Plants were grown in the greenhouse to sample caryopses or harvest seeds. Seedlings at 3 to 4 wk old were transplanted into pots at 1 plant/pot, and the pots filled with a mixture of medium (Sunshine Mix ^#^1) and clay soil in a 2:1 (v/v) ratio. Day/night temperatures were set at 27/21 °C with an Argus Controls (Surrey, Canada) system, and supplementary light (SolarSystem 550 Premium LED Grow Lights, CA, USA) was used to ensure >12 h daylengths. Plants were tagged for flowering date when the first panicle of a plant emerged from the leaf sheath. Seeds (filled spikelet) were harvested at 40 d after flowering, air-dried for 3 d on a bench in the greenhouse, and stored in a −20 °C freezer to maintain the status of primary dormancy.

### Genotyping and sequencing

Plants were genotyped with polymorphic markers (Supplementary Table 2) to delimit the *Rc-* and *Pb*-introgression segments in the ILs or to identify 9 genotypes for the *Rc/rc* and *Pb/pb* alleles in the F_2_ population. To identify point mutations reported for the *pb* allele (Wang and Shu 2007; Zhu et al. 2017), the 4 pairs of PCR primers (Supplementary Table 2) were used to sequence DNA fragments at *OsB1* or *OsB2* from IL_rcpb_ and IL_rcPb_. The primers were designed based on the reference genome sequence (Kawahara et al. 2013). Methods for DNA preparation, PCR, electrophoresis, and sequencing were same as described in Gu et al. (2011).

### Histological analysis

Panicles emerging on the same date were selected to sample developing caryopses. Florets were marked with a red/dark dot on the hull and collected at 5, 10, or 15 d post-anthesis (DPA) to isolate intact caryopses. The fresh caryopses were fixed in a formalin aceto-alcohol solution before being stored in a refrigerator (4 °C). The fixed caryopses were dehydrated, rehydrated, and embedded in Paraplast (Sigma-Aldrich, St. Louis, MO, USA) using the methods described in Luna (1968). The embedded tissues were sectioned at 8 to 10 µm thick using a rotary microtome (Olympus CUT 4060E, Bartlett, TN, USA). The sectioned tissues were stained with hematoxylin and eosin (Sigma) and imaged using an upright compound microscope (Olympus AX70).

### Flavonoid extraction and measurement

Caryopses at 5 or 40 DPA were sampled from the IL plants on the same days and the samples stored in a freezer (−20 °C). Flavonoid extraction was performed for 3 replicates using the optimized protocols (Shi and Xie 2010; Peng et al. 2012; Yuzuak et al. 2018). A sample of 50 caryopses were ground into powder in liquid nitrogen. The powder of 0.1 g was suspended with 1.0 mL buffer [0.5% HCl in methanol:diH_2_O (50:50, v/v)] in a 1.5-mL Eppendorf tube at room temperature. The tube was vortexed for 45 s and centrifuged at 9,391 rcf for 10 min. The supernatant was transferred into a 1.5-mL tube. This step was repeated twice. To remove chlorophyll and nonpolar lipids, the extraction was mixed with 0.2 mL chloroform, and the mixture vortexed for 45 s and centrifuged at 9,391 rcf for 5 min. The resulting upper methanol:water phase containing flavan-3-ols and anthocyanins was transferred to a new tube and dried for 2 h using a SpeedVac Concentrator connected to Refrigerated Condensation Trap. The remaining pellets were dissolved in 0.5 mL of acidified methanol (0.1% HCl).

The anthocyanin and flavan-3-ol profiles were analyzed using the protocol outlined in He et al. (2017) and Yuzuak et al. (2018) and a LC-qTOF-MS/MS system (Agilent, Santa Clara, CA, USA). The mobile phase solvents include 1% acetic acid in water (solvent A: 1% HPLC grade acetic acid in LC-MS grade water) and 100% acetonitrile (solvent B) (LC-MS grade). A gradient elution system was optimized with the 2 mobile solvents to separate generated phenolics in an analytical column (Agilent Elipes XDB-C18, 250 × 4.6 mm, 5 µM, 25 °C). The gradient solvent system was composed of solvents A to B in ratios: 95:5 (0 to 5 min), 95:5 to 90:10 (5 to 10 min), 90:10 to 85:15 (10 to 15 min), 85:15 to 45:55 (15 to 45 min), 45:55 to 25:75 (45 to 50 min), and 25:75 to 95:5 (50 to 60 min). After the last gradient step, the column was equilibrated and washed for 10 min with solvents A:B (95:5). The ﬂow rate was 0.4 mL/min. The injection volume of the samples was set at 5.0 µL. The drying gas ﬂow and nebulizer pressure were set at 12 L/min and 50 psi, respectively. Metabolites were ionized with a negative mode. The mass spectra were scanned from 100 to 3,000 m/z. The acquisition rate was 3 spectra/s. Other MS conditions optimized include fragmentor at 150 V, skimmer at 65 V, OCT 1 RF Vpp at 750 V, and collision energy at 30%. The authentic (−)-epicatechin, (+)-catechin, (−)-epigallocatechin, (+)-gallocatechin, and cyanidin-3-glucoside (Yuzuak et al. 2018) were used as references.

### Seed dormancy assessment

Seed primary dormancy was evaluated for the IL and F_2_ plants by germination testing. Seed samples from the freezer were moved to the room conditions of 23.7 ± 0.10 °C and 28.5% ± 5.2% relative humidity (RH) for a given period (0 to 14 d, depending on experiments) of after-ripening (DAR) to best display genotypic variation in germination percentage (GP) or index (GI). For the ILs, seeds from individual plants of the same line were bulked and 9 samples used for germination testing. For the F_2_ plants, 3 samples of seeds from a plant at 14 DAR were used for the testing. Each sample of ∼60 seeds was distributed in a 9-cm Petri dish, which was lined with a piece of filter paper and soaked with 8 mL water, and the samples were placed in sealed containers to maintain a humid environment. Germination testing was conducted in an incubator (Thermo Scientific Precision 818) set at 30 °C and no illumination for 7 d. Germinated seeds (radicle protrusion > 3 mm) were counted daily under bright white, fluorescent tube lights from the second d for the ILs or at the third, fifth, and seventh days of imbibition (DOI) for the F_2_ population. GP at day i (GP_i_) were calculated as,


(1) $$GP_{i}=\sum \left(n_{i}/N\right)$$

where n_i_ is the number of seeds germinated on day i and *N* is the total number of seeds in a sample. GI is a weighted GP average calculated as


(2) $$\begin{array}{r} \begin{array}{c} \mathrm{GI}=100\times (n_{2}\times 6+n_{3}\times 5+n_{4}\times 4+n_{5}\times 3+n_{6}\times 2+n_{7}\times 1)\\ \;/(N\times 7)\mathrm{for}\;\mathrm{the}\;\mathrm{ILs},\mathrm{or}\\ \;\;\;=100\times (n_{3}\times 5+n_{5}\times 3+n_{7}\times 1)/(N\times 7)\mathrm{for}\;\mathrm{the}\;F_{2}\;\mathrm{plants} \end{array} \end{array}$$

where the numbers 1 to 6 are weights applied to n_i_ at 2 to 7 DOI. Means of the 3 samples from an F_2_ plant were used for quantitative genetic analysis.

### Germination or imbibition velocity assessment

Three experiments (Exp.) were conducted for dormancy-released seeds from the 4 ILs. Seed samples were air-dried under the room conditions for 30 d (Exp. 1) or dried at 40 °C in an oven for 14 d (Exp. 2) to break the dormancy. Ten samples from a line were germinated under the above-stated conditions to evaluate germination velocity.

The third Exp. was designed to identify germination distribution patterns and genic effects on imbibition speed. The germination process can be divided into 3 phases, imbibition (I), plateau (II), and resumption of water uptake (III), based on the seed water content (Bewley et al. 2013). Seed samples were after-ripened at the room temperature for >60 d to break the dormancy. Five 100-seed samples from each line were weighed (w_0_) before soaking in a 9-cm Petri dish with 10 mL water. The soaked samples were placed in the incubator set at 30 °C, 100% RH and dark conditions, and weighed (w_i_) at time points (i): 0.5 h, and every 2 h from 2 to 12 h, every 4 h from 12 to 60 h, or every 12 h from 60 to 96 h. To weigh the samples, seeds were transferred to 50-mL tubes holed in the bottom and centrifuged at 145 rcf for 1 min to remove the water on the hull. The water content at the ith point (wc_i_) was calculated as (w_i_ − w_0_)/w_0_.

### Quantitative real-time polymerase chain reaction (qRT-PCR)

qRT-PCR was used to evaluate the transcript abundance of genes reported for the flavonoid biosynthesis in rice (Supplementary Table 2; Xia et al. 2021). The IL plants were grown in a greenhouse to sample caryopses at 5 DPA. The pericarp tissue was isolated from the caryopses by squeezing out the embryo and endosperm. Two or 3 biological replicates of 50 pericarps were collected in liquid N2 and stored in a freezer (−80 °C). Total RNAs were extracted using the RNeasy Plant Mini Kit (Qiagen, Hilden, Germany) and treated with RNase-Free DNase (Invitrogen, Lohne, Germany). The RNA quality and purity were assessed using NanoDrop Spectrophotometer (ThermoFisher Scientific, Wilmington, DE, USA) and checked by electrophoresis on 1% agarose gel. A sample of 1 μg RNA and the high-capacity cDNA reverse transcription kit (Applied Biosystem, Waltham, MA, USA) were used to synthesize cDNAs. Transcription abundance for the selected genes was quantified with the cDNAs, primers (Supplementary Table 2), and SYBR green super mix (Promega, Madison, WI, USA) using the 7900HT Fast Real-Time PCR system (Applied Biosystem, Foster, CA, USA). *ACTIN* was used as an internal control. Cycle threshold (Ct) data for the tested and control genes from the same experiments were calculated for the 2^−ΔCt^ values to estimate relative expression levels (Livak and Schmittgen 2001).

### Quantitative genetic analysis

The 4 ILs varied in pericarp color with seed development times (T). Thus, the phenotypic variance for each type of the flavonoid chemicals in the digenic (*Rc/rc* and *Pb/pb*) system was partitioned into its components using the multiple linear regression model:


(3) $$y_{ijkl}=\mu +a_{1}x_{i}+a_{2}x_{j}+i_{a1a2}x_{ij}+\tau z_{k}+I_{a1\tau }w_{ik}+I_{a2\tau }w_{\;jk}+\varepsilon_{ijkl}$$

where the dependent variable *y_ijkl_* is the flavonoid content for a genotype of the *Rc* (*i*) and *Pb* (*j*) loci at the time point *k* in the *l*th replicate; *μ* is the model mean or background effect; *x_i_* and *x_j_* are independent variables for the genotypes at loci *i* or *j*, with *i* (*j*) coded as −1 for *rcrc* (*pbpb*) or 1 for *RcRc* (*PbPb*), and *a_1_* and *a_2_* are the partial regression coefficients or additive effects of the *Rc* and *Pb* loci, respectively; *x_ij_* is the variable for an epistatic component of the 2 loci and coded as the product of *x_i_* and *x_j_*, and *i_a1a2_* is the coefficient or epistatic effect; *z_k_* is the variable for the T component, with *k* = 0 for 5 or 1 for 40 DPA, and *τ* is the coefficient or main effect of T; *w_ik_* and *w_jk_* are variables for the international components of *z_k_* with *x_i_* or *x_j_*, and coded as the products of *z_k_* and *x_i_* or *x_j_*, and *I_a1τ_* and *I_a2τ_* are the coefficients or international effects; and *ε_ijkl_* is the error term.

Germination data from the F_2_ population were analyzed using a 2-locus additive-dominance-epistasis model:


(4) $$\begin{array}{rl} y_{ijk}= & \;\mu +a_{1}x_{i}+d_{1}z_{i}+a_{2}x_{j}+d_{2}z_{j}+i_{a1a2}w_{xixj}+i_{a1d2}w_{xizj}+i_{d1a2}w_{zixj}\\ & +i_{d1d2}w_{zizj}+\varepsilon_{ijk} \end{array}$$

where *y_ijk_* is the GP or GI of the *k*th plant from the genotype for the *Rc* (*i*) and *Pb* (*j*) loci; *µ*, *x_i_*, *x_j_*, *a_1_*, and *a_2_* are defined in Eq. 3, with *i* (*j*) = −1 for the *rcrc* (*pbpb*), 0 for the *Rcrc* (*Pbpb*), or 1 for the *RcRc* (*PbPb*) genotype; *z_i_* and *z_j_* are variables for the dominance components of the *i* or *j* locus, with *i* (*j*) = 0.5 for *Rcrc* (*Pbpb*) or −0.5 for the homozygous genotypes, and *d_1_* and *d_2_* are the partial regression coefficients or dominance effects of the loci; *w_xixj_*, *w_xizj_*, *w_zixj_* and *w_zizj_* are variables for the 4 types of epistatic interactions between the 2 loci for the additive or dominance components, with each coded as the product of codes for the components indicated in the subscripts; *i_aa_*, *i_ad_*, *i_da_*, and *i_dd_* are partial regression coefficients for the epistatic components or epistatic effects; and *ε_ijk_* is the residual of the model.

The datasets for germination velocity and seed water content at each of the time points (wc_i_) were analyzed using the 2-locus model:


(5) $$y_{ijk}=\mu +a_{1}x_{i}+a_{2}x_{j}+i_{a1a2}w_{ij}+\varepsilon_{ijk}.$$

The model Eq. 5 was modified from Eq. 4 by removal of the variables for the dominance component, as it is absent in a set of homozygous genotypes. The additive and dominance variables in the models Eqs. 3–5 were coded to comply with the orthogonal property for a proper estimation of genetic effects (Zeng et al. 2005). Analyses of correlation, variance or linear regression were performed using the SAS GLM or REG programs (SAS Institute 2020). A stepwise selection at a significant level of 5% was used to retain component variables in the models.

## Results

### Morphological and genotypic differences among the 4 ILs

The 4 lines differed in caryopsis color, but little in flowering time and plant morphologies. The time to flowering was 78.8 to 79.1 d, the plant height was 83.5 to 84.0 cm at flowering, and the spikelet was identical for straw-colored, non-awned hulls at maturation (Supplementary Fig. 1a and b). The caryopses were colored for the pericarp, not the endosperm (Supplementary Fig. 1c).

**Fig. 1.**
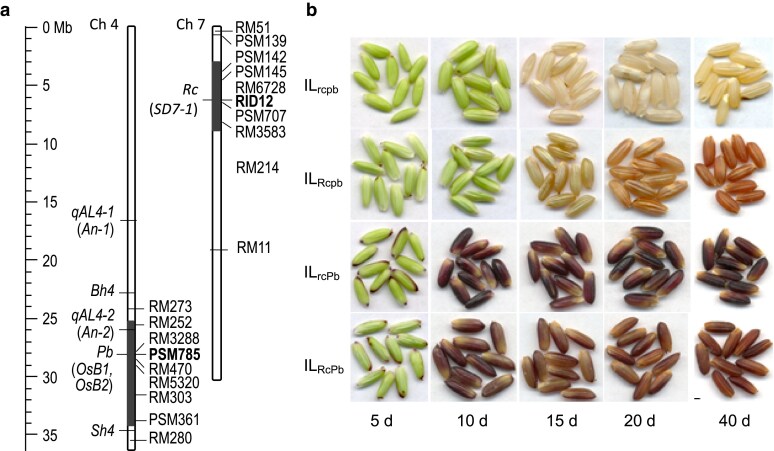
Genotypic and phenotypic differences among the 4 ILs for the *Rc/rc* and *Pb/pb* alleles. a) Map positions of the *Rc* and *Pb* loci on chromosomes (Ch) 4 or 7. Each Ch is labelled with markers (right) and genes (left) to delimit the segments from donors (filled) in the recipient (IL_rcpb_) background (open). Genes/QTL for the black hull (*Bh4*), awn length (*An-2/qAL4-2*), and seed shattering (*Sh4*) or dormancy (SD) are aligned to the reference genome sequence in Mb (Kawahara et al. 2013). b) Developmental patterns of seed pigment traits. The ILs are homozygous for the alleles indicated in the subscripts. The images show caryopses at 5 to 40 d post-anthesis. Bar = 1 mm.

The 2 introgression segments were delimited to physical regions of <10 Mb. The *Rc*- and *Pb*-containing segments were about 6 and 8 Mb, respectively, based on 10 markers on each of the chromosomes (Fig. 1a). The *rc* allele from the recipient contains a 14-bp deletion (Supplementary Fig. 1d), a loss-of-function mutation marked by RID12 (Sweeney et al. 2006).

The *Pb*-introgression is flanked by the black hull (*Bh4*) and seed shattering (*Sh4*) loci and encompasses the *OsB1*, *OsB2*, and awn (*An-2*) genes (Fig. 1a). The *Pb* and *An-2* loci are linked on an interval of ∼2 Mb. The phenotypes of straw-colored, non-awned hulls (Supplementary Fig. 1b) suggest that the *Pb*'s donor and recipient parents carry mutant *bh4* and *an-2* alleles. Genotyping with PSM785 detected a 30-bp deletion in the marker allele from IL_Rcpb_ and IL_rcpb_ (Supplementary Fig. 1d and e). This deletion mutation is present in the fifth intron of *OsB1* (Supplementary Fig. 2a). Sequencing the 4 fragments from IL_rcpb_ and IL_rcPb_ (Supplementary Fig. 2a) detected 3 mutations at *OsB1* and 1 mutation at *OsB2* (Supplementary Fig. 2b and c). All 3 mutations are in the intron regions (Supplementary Fig. 2a), suggesting that *Pb*'s functional mutation(s) are present in the other regions, including the promoter of *OsB2* (Oikawa et al. 2015). The phenotypic and genotyping data support that the 4 lines contain functionally differentiated *Rc/rc* and *Pb/pb* alleles in a purified genetic background.

### Four developmental patterns for the 2 seed pigment traits

Distinct pigmentation patterns were observed on caryopses from the 4 ILs. Early developing caryopses are green due to the presence of chlorophylls in the pericarp lower-middle layer in rice (Hoshikawa 1997). The 2 *Pb-*absent lines lost the chlorophylls at 15 DPA, when the pericarp became white in IL_rcpb_ or brown in IL_Rcpb_; with seed development, the coat color turned to light brown in the double mutant at 40 d, or light red at 20 d and bright red at 40 d in the *Rc*-present line (Fig. 1b). In contrast, the 2 *Pb-*present lines started to show purple color on the stylar and dorsal areas at 5 d, with the pigmented area smaller in IL_RcPb_ than in IL_rcPb_; then the pigment was extended to the entire surface of the caryopses, with the color darker in IL_rcPb_ (dark purple) than in IL_RcPb_ (red purple) from 10 to 40 d (Fig. 1b). The 4 patterns confirmed the regulatory role of *Rc* and *Pb* in the development of red and purple pericarp traits, respectively, and suggested that *Rc* and *Pb* may interact to influence the pigment biosynthesis.

### Red and purple pigments were synthesized in the same cells

**Fig. 2.**
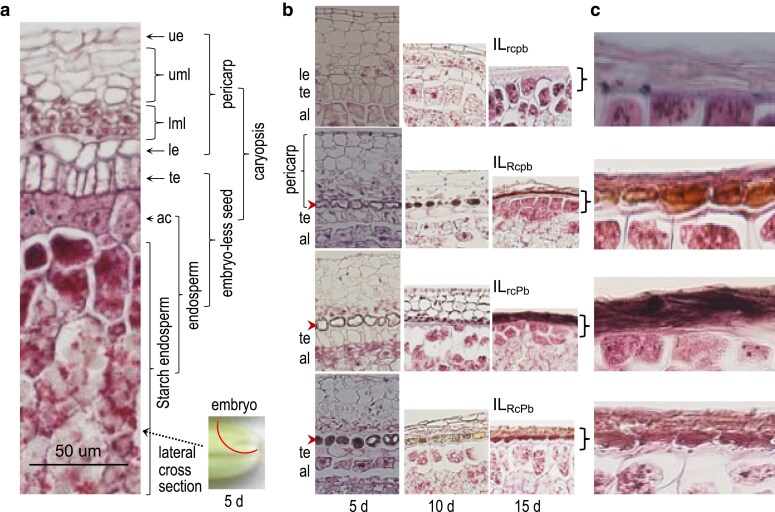
Developmental and compartment patterns of seed pigment traits. a) Structures of a 5-d caryopsis. The images show a lateral cross-section away from the embryo (the area circulated by a dotted line) to display the endosperm, testa (te), and pericarp tissues in IL_rcpb_. The pericarp is separated from the aleurone layer (al) by a tetsta (te), and it consists of upper (ue) and lower (le) epidermises, and upper (uml) and lower (lml) middle layers. b) Pericarp histological changes and pigment compartment patterns. The images show lateral cross-sections of 5-, 10-, or 15-d caryopses from the 4 ILs (Fig. 1b), each aligned with the le layer to compare genotypic differences in the compartment pattern. The arrowheads indicate the pigments covering the entire le cells in IL_Rcpb_ and IL_RcPb_ or distributed in the lignified cell wall area in IL_rcPb_ at 15 d.

The pericarp at 5 DPA was well-developed and biologically active. The coat tissue was separated from the endosperm by a layer of testa cells and the lateral section differentiated into the upper (ue) and lower (le) epidermises of a single-cell layer and the upper- and lower-middle layers of multicell lines (Fig. 2a). Pigmentation was visualized in the le cells in IL_Rcpb_, IL_rcPb_, and IL_RcPb_, with the hematoxylin stain started from the peripheral area at 5 d and extended to the entire cell frame at 10 d (Fig. 2b). The stain intensity became stronger at 15 DPA, when the middle layers largely disappeared, and the low layer varied in morphology with the genotypes. For example, the stains covered the le cells evenly in IL_Rcpb_, spread along the lignified cell wall area of the lower-middle layer in IL_rcPb_, or distributed unevenly over the le cells with the peripheral area thicker than the central area in IL_RcPb_. The distribution patterns suggest that the *Rc-*controlled pigments are compartmented within the cell frame while the *Pb-*controlled pigments concentrated in the lignified wall area in the dead fruit coat.

### The *Rc* and *Pb* genes regulated different subgroups of flavonoids

Five types of flavonoid chemicals were detected in 5- and 40-d caryopses from the ILs. The chemicals belong to the flavan-3-ol or anthocyanins (AC) subgroups (Table 1). The flavan-3-ols are catechin (CA), epicatechin (EC), dimeric CA procyanidin B3 (PB3), and dimeric EC procyanidin B2 (PB2). CA was present in the 4 lines but abundant in the 2 *Rc*-present lines (10 μg/mg); whereas EC, PB2, and PB3 were present only in the 2 lines, and the 3 together accounted for ∼1/3 of the CA content in IL_Rcpb_ or IL_RcPb_. Modeling (Eq. 3) analysis identified significant effects on each of the flavan-3-ols for the *Rc*-additive (*a_1_*), development time (*τ*), and interactional (*I_τa1_*) components (Supplementary Table 3). The 3 effects were positive or acted to increase the flavan-3-ols. Of the 3 effects, *a_1_* accounted for 77%, 81%, 86%, and 88% of the total variances for PB2, EC, CA, and PB3, respectively, and both *τ* and *I_τa1_* together contributed 9% (PB3) to 19% (PB2) to the totals. The epistatic effect *i_a1a2_* was not significant, suggesting that *Rc* is independent of *Pb* in regulating the flavan-3-ol biosynthesis.

**Table 1.** Summary of statistics for flavonoid contents in 5- and 40-d caryopses from the 4 lines (Fig. 1b).

Line	Time (d)	CA (µg/g)	PB3 (µg/g)	EC (µg/g)	PB2 (µg/g)	AC (µg/g)
IL_rcpb_	5	1.0 (0.3)	-	-	-	-
	40	248 (53)	-	-	-	-
IL_Rcpb_	5	5,627 (262)	1,673 (84)	106 (18)	209 (26)	-
	40	10,167 (637)	3,072 (168)	202 (11)	510 (36)	-
IL_rcPb_	5	17.9 (4.3)	-	-	-	2,331 (128)
	40	9.7 (3.3)	-	-	-	3,271 (231)
IL_RcPb_	5	6,306 (239)	2,154 (107)	125 (16)	297 (30)	125 (13)
	40	10,149 (768)	3,062 (215)	234 (9)	555 (26)	3,326 (168)

Data shown are means (SE) of 3 replicates for anthocyanins (AC), catechin (CA), epicatechin (EC), or procyanidins B2/B3 (PB2/PB3). A dash (-) indicates not detectable.

Anthocyanins were present only in the 2 *Pb*-present lines (Table 1). The AC content at 5 d was ∼17 times lower in IL_RcPb_ (0.13 mg/g) than in IL_rcPb_ (2.3 mg/g), which was consistent with the morphological difference in the purple-pigmented area on the caryopses between the lines (Fig. 1b). Modeling analysis identified significant effects for the *Pb-*additive (*a_2_*), *τ*, *i_a1a2_*, and interactional (*I_τa2_*) components, with the epistatic effect *i_a1a2_* reducing and the other effects increasing the AC content (Supplementary Table 3). Of the 4 components, *I_τa2_* accounted for a majority (63%) of the total variance, and the others explained ∼4% (*i_a1a2_*) to 12% (*τ*) of the total. These estimates suggest that the *Pb*-regulated AC biosynthesis is strongly influenced by the seed development times, both directly (*τ*) and indirectly (*I_τa2_*), as well as *Rc*. The epistatic mechanism appeared to limit the AC biosynthesis at the early stage of seed development (Fig. 1b).

The catechin content was low (<0.25 mg/g on average) in IL_rcpb_ and IL_rcPb_ (Table 1). As the only flavonoid chemical detected from the double mutant, the CA oxidation accounted for the light-brown color on the 40-d caryopses (Fig. 1b). The low level of CA in these 2 lines suggests the presence of additional CA-regulatory gene(s) in the genetic background.

### Shared or specific flavonoid biosynthesis pathways regulated by *Rc* and *Pb*

**Fig. 3.**
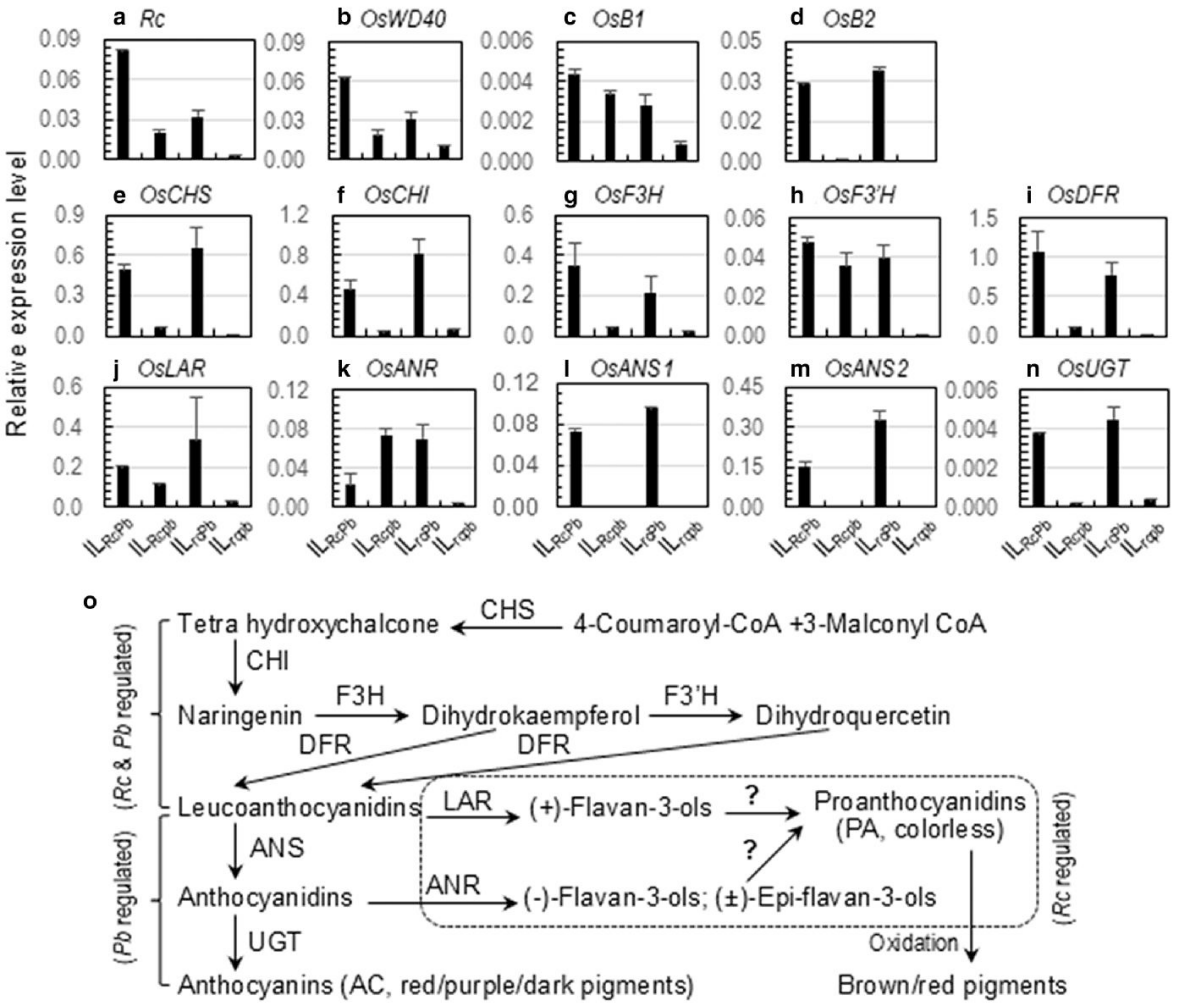
Genotypic differences in transcription levels of flavonoid biosynthesis genes in 5-d pericarps from the 4 ILs (Fig. 1b). a to d) Regulatory genes *Rc* a), *OsWD40* b), *OsB1* c) and *OsB2* d). e to i) Structural genes *OsCHS* e), *OsCHI* f), *OsF3H* g), *OsF3*′*H* h), and *OsDFR* i) for leucoanthocyanidin (LAC) synthesis. j to n) Structural genes *OsLAR* j) and *OsANR* k) for PA biosynthesis or *OsANS1* l), *OsANS2* m), and *OsUGT* n) for AC biosynthesis. o) The LAC, PA, or AC synthesis pathways. The regulatory and structural genes are listed in Supplementary Table 2. Columns (bars) indicate means (SE) of 2 or 3 replicates. The pathways were modified from Xie and Dixon (2005) and grouped based on the transcriptional and metabolic data (Table 1). AC, anthocyanins; ANR, anthocyanidin reductase; ANS, ANTHOCYANIDIN SYNTHASE, which is encoded the paralogs OsANS1 or OsANS2 in the rice genome; LAR, leucoanthocyanidin reductase; PA, proanthocyanidins; UGT, UDP-glucosyltransferase. Question marks indicate the steps unknown for biochemical mechanism.

Four regulatory and 10 structural genes were quantified for relative expression levels in the 5-d pericarps. Of the regulatory genes, 3 (*Rc*, *OsWD40*, and *OsB1*) were expressed in the AC- and/or PA-containing lines, with the expression level higher in IL_RcPb_ than that in IL_Rcpb_ or IL_rcPb_ (Fig. 3a to c). Different from *OsB1*, its paralog *OsB2* was expressed specifically in the 2 AC-containing lines (Fig. 3d), suggesting that the OsB2 TF may regulate AC synthesis specifically.

Five of the structural genes encode enzymes catalyzing the early steps of flavonoid biosynthesis for the leucoanthocyanidin (LAC) intermediate product. Of the 5 genes, *OsF3′H* was expressed at a similar level in the AC- and/or PA-containing lines, and the others (*OsCHS*, *OsCHI*, *OsF3H*, and *OsDFR*) had a lower expression level in IL_Rcpb_ than in IL_rcPb_ and IL_RcPb_ (Fig. 3e to i). The lower expressions in IL_Rcpb_ suggest that the enzymes could be also encoded by other paralogs in the genome, or *Rc* and *OsB2* may regulate the shared pathways with dissimilar mechanisms.

The 5 structural genes encode enzymes catalyzing the late steps for the PA or AC biosynthesis. Of the 5, 2 (*OsLAR* and *OsANR*) were expressed in the AC- and/or PA-containing lines (Fig. 3j and k), and the others (the *OsANS1* and *OsANS2* paralogs and *OsUGT*) expressed specifically in the AC-containing lines (Fig. 3l to n). Based on the metabolic (Table 1) and transcriptional (Fig. 3e to n) profiles, the 10 structural genes can be divided into 3 groups associated with the LAC, AC, and PA productions, respectively, in the digenic system (Fig. 3o).

### Associations of the *Rc* and *Pb* loci with seed primary dormancy

**Fig. 4.**
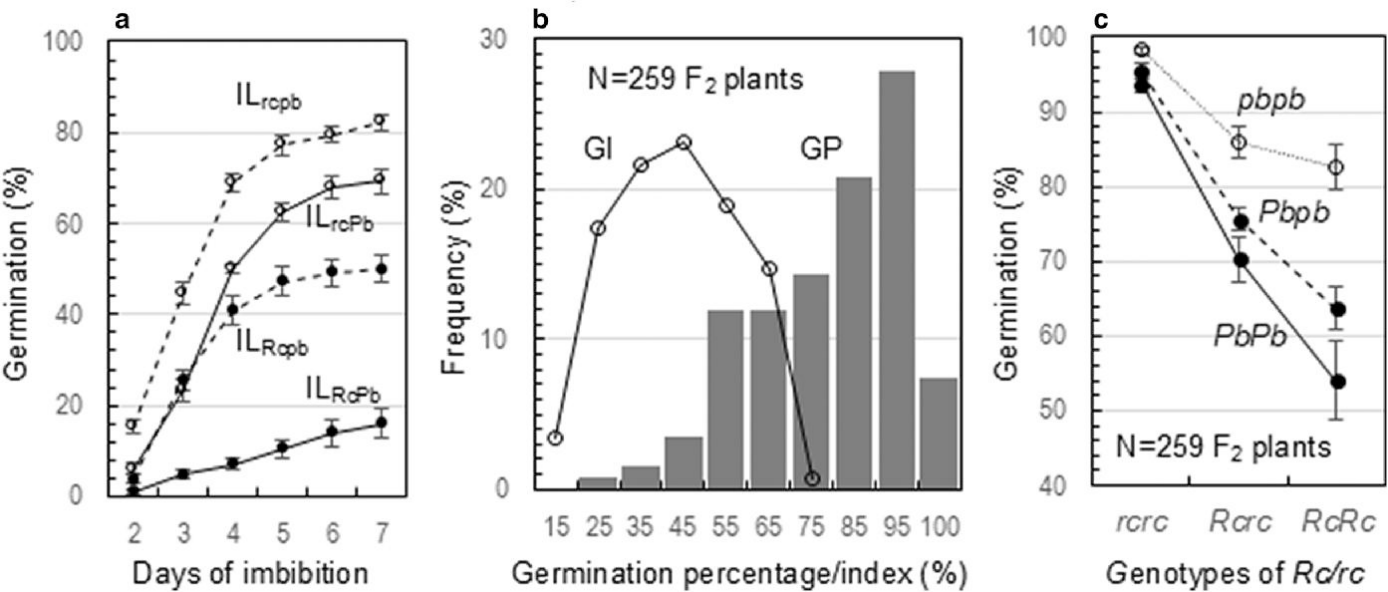
Phenotypic and genotypic variations in seed dormancy associated with the *Rc* and *Pb* loci. a) Germination distribution patterns for the 4 ILs. The ILs are homozygous for the *Rc/rc* and *Pb/pb* alleles indicated in the subscripts. Germination was evaluated for dry seed samples without an after-ripening treatment. Circles (bars) indicate means (SE) of 9 samples at 2 to 7 d of imbibition. b) Frequency distributions of germination percentage (GP) and index (GI) in an F_2_ population of N plants. Germination was evaluated for seed samples after 7 d of after-ripening. c) Interaction pattern of the 2 loci. Circles (bars) indicate genotypic means (SD) for the GP data in B, and the lines are used to visualize the interactional pattern.

The association was first observed in the 4 ILs. They differed from each other in the pattern of germination distribution from 2 to 7 d of imbibition (DOI), and the mean GP at 7 d or GP7 (Eq. 1) was 16%, 50%, 69%, and 82% for IL_RcPb_, IL_Rcpb_, IL_rcPb_, and IL_rcpb_, respectively (Fig. 4a). The variances in GP and GI were partitioned into additive effects of the *Rc* (*a_1_*) and *Pb* (*a_2_*) loci and the *i_a1a2_* epistasis (Table 2). The 3 effects were all negative, indicating that the *Rc* and *Pb* alleles acted to reduce germination or enhance dormancy. Compared with *Pb*, *Rc* had a greater effect on reduction of germination and contributed (R^2^) about 30% more to the total variances in GP or GI. Of the 3 components, the epistasis was lowest in effect size and contributed least (1% to 4%) to the variances.

**Table 2.** Summary of estimates for genic effects of *Rc* and *Pb* on seed dormancy in the IL or F_2_ systems.

System	SD^a^	Estimate^b^	*µ*	*a_1_*	*d_1_*	*a_2_*	*i_a1a2_*	Total
ILs	GP	Effect (%)	54.4	−21.3	-	−11.7	−5.2	
		Pr. > F	<0.0001	<0.0001	-	<0.0001	0.0007	
		R^2^		66.7	-	20.1	4.0	90.8
	GI	Effect (%)	32.7	−13.4	-	−9.0	−2.6	
		Pr. > F	<0.0001	<0.0001	-	<0.0001	0.0009	
		R^2^		63.2	-	28.2	2.5	93.9
F_2_	GP	Effect (%)	78.6	−15.0	−3.9	−8.2	−6.2	
		Pr. > F	<0.0001	<0.0001	0.0173	<0.0001	0.001	
		R^2^		31.7	2.5	9.1	1.3	44.6
	GI	Effect (%)	43.1	−12.3	−5.7	−9.7	-	
		Pr. > F	<0.0001	<0.0001	<0.0001	<0.0001	-	
		R^2^		31.5	3.8	19.2	-	54.5

^a^SD, seed primary dormancy evaluated by germination percentage (GP in Eq. 1) and index (GI in Eq. 2).

^b^Estimates for the background (*µ*), *Rc-*(*a_1_*) and *Pb-*(*a_2_*) additive, *Rc-*dominance (*d_1_*), and *a_1_*–*a_2_* epistatic (*i_a1a2_*) effects in Eq. 4 or Eq. 5. Negative values indicate that the genic effects acted to enhance SD, a dash (-) indicates not significant at the probability (Pr.) level of 0.05, and R*^2^* was the proportion of the variance explained by the effect.

The association was also observed in the F_2_ population. Nine *Rc/rc* and *Pb/pb* genotypes were identified from a population of ∼300 F_2_ seedling, with frequencies varying from 4% for *RcRcPbPb* to 30% for *RcrcPbpb*. The 9 genotypes of caryopses displayed 4 colors, i.e. red purple, purple, red, and white, like those for the 4 ILs (Fig. 1b). Thus, *Rc* and *Pb* are completely dominant over *rc* and *pb*, respectively, for the pigment traits. Dormancy was evaluated for 259 F_2_ plants with enough seeds for duplicated germinating testing. The F_2_ plants were distributed over the 20% to 100% range for GP7 or the 10% to 80% range for GI (Fig. 4b). Four genic effects (*a_1_*, *a_2_*, *i_a1a2_*, and *d_1_ Rc*'s dominance) were detected for GP7, but only the *a_1_*, *d_1_*, and *a_2_* effects were significant for GI (Table 2). All the genic effects were negative or acted to reduce germination. The dominance ratio *d_1_*/*a_1_* was 0.26 for GP or 0.46 for GI, indicating that *Rc* was partially dominant over *rc* for the dormancy trait. The epistasis for GP was characterized by the variation among the *Pb/pb* genotypes increased with the number of a functional allele at the *Rc* locus (Fig. 4c).

### Associations of the *Rc* and *Pb* loci with germination velocity

**Fig. 5.**
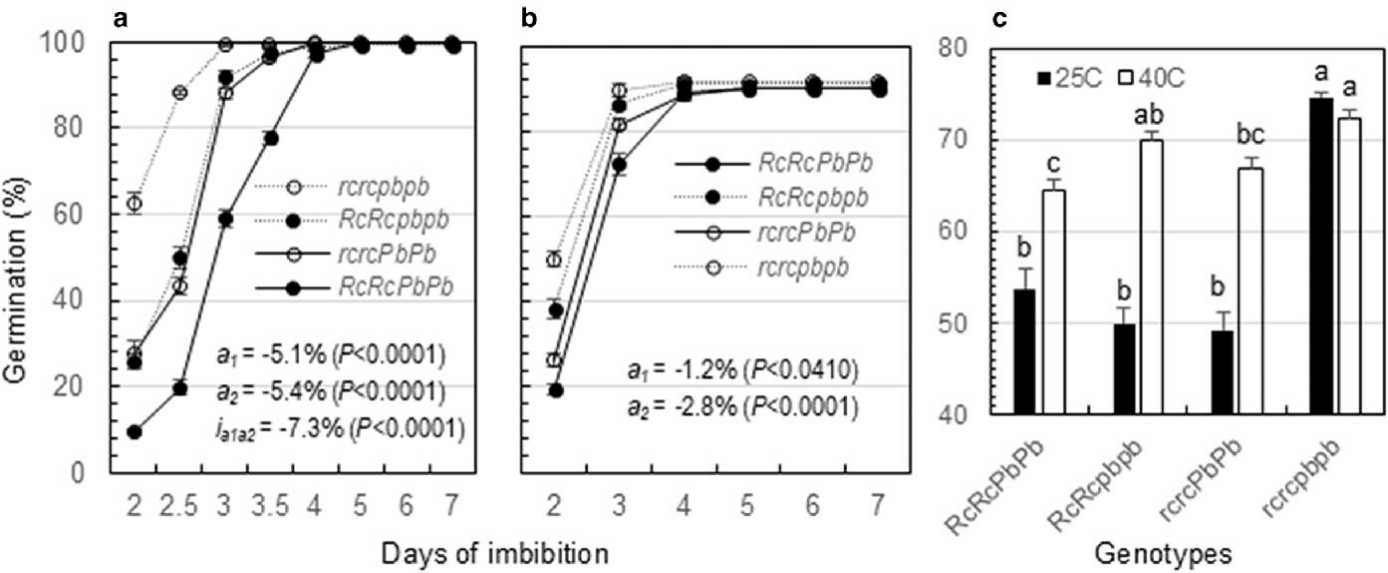
Genotypic differences in germination velocity among the 4 ILs. a and b) Germination distribution patterns. Germination was evaluated for seed samples stored under the room conditions for 30 d a) or heated at 40 °C for 14 d b) to break the dormancy. The circles (bars) indicate means (SE) of 5 a) or 10 b) replicates. The *Rc-*(*a_1_*) and *Pb-*(*a_2_*) additive and their epistatic (*i_a1a2_*) effects were evaluated for GI (Eq. 2) using the model in Eq. 5. c) Germination indexes for the data in a) or b). The letters (a to c) indicate significant differences at *P* < 0.001.

The 2 loci were also associated with germination velocities of seeds treated with 30 d of after-ripening (Exp. 1) or 14 d of heating at 40 °C (Exp. 2). The 4 lines reached 99% to 100% germination in Exp. 1 or 90% to 92% germination in Exp. 2 at 5 DOI (Fig. 5a and b), indicating that the dormancy with the samples was broken by the treatments. However, the 4 ILs differed in GP at 2 and 3 DOI in both experiments, and the weighted GP average (GI in Eq. 2) varied from 49% (IL_rcPb_) to 75% (IL_rcpb_) in Exp. 1 or from 64% (IL_RcPb_) to 72% (IL_rcpb_) in Exp. 2 (Fig. 5c). Genic effects of the *Rc* and *Pb* loci on GI were significant for the *a_1_*, *a_2_*, and *i_a1a2_* components in Exp. 1 or for *a_1_* and *a_2_* in Exp. 2. All the component effects were negative or acted to reduce GI by 5% to 7% in Exp. 1 (Fig. 5a) or 1% to 3% in Exp. 2 (Fig. 5b).

**Fig. 6.**
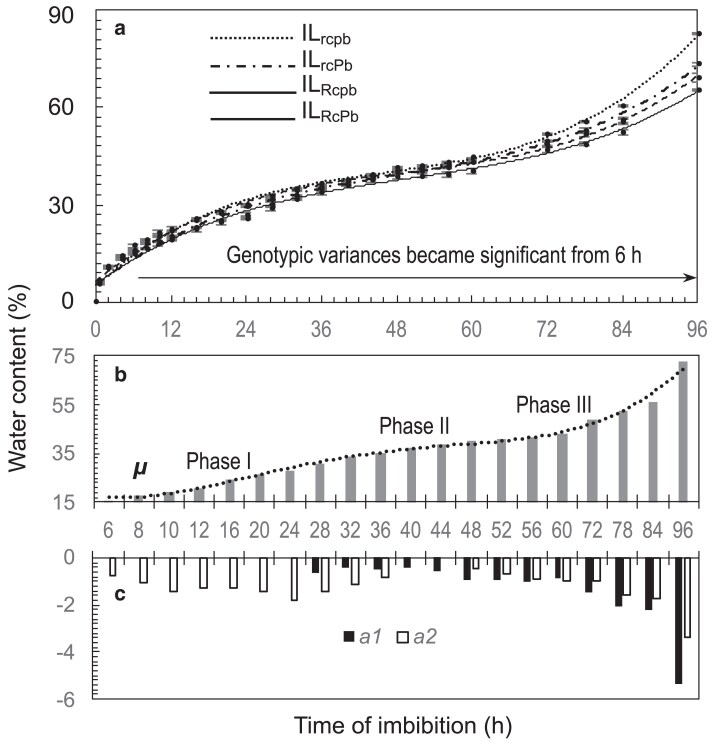
Modeling component effects of the *Rc* and *Pb* loci on seed imbibition speed. a) Temporal distributions of seed water content for the 4 ILs at 0 to 96 h of imbibition. Circles (bars) indicate means (SE) of five 100-seed samples after the dormancy was released. The horizontal line indicates the period when 1 or more of the effects were significant (Supplementary Table 4). b) Temporal distribution for the model mean (*µ*) in Eq. 3. The columns are the *µ* values in Supplementary Table 4, and the dotted line traces imbibition speeds during the imbibition (I), plateau (II), and post-germination (III) phases of the germination process. c) Temporal distributions for additive effects of the *Rc* (*a_1_*) and *Pb* (*a_2_*) loci. Columns are the estimates in Supplementary Table 4.

Genotypic differences in seed water content were observed in the imbibition experiment. The difference increased with the imbibition time, as shown by the temporal distribution patterns from 0 to 96 h after soaking (Fig. 6a). The variances in wc at each of the time points were partitioned into the background (*µ*), additive (*a_1_* and *a_2_*) and epistatic (*i_a1a2_*) effects (Supplementary Table 4). The *µ* estimates are independent of the genic effects (Eq. 5) and thus were used to model the lengths and imbibition speeds for each of the 3 phases. Phases I (imbibition) and II (plateau) lasted for ∼30 and ∼20 h, respectively, and Phase III started at ∼50 h (Fig. 6b). The genic effects became significant at 6 h, the effect sizes varied with the genes and times, and the genic effects accounted for 23% to 90% of the total variances in wc at 6 to 96 h (Supplementary Table 4). For example, *a_2_* at 6 h and *a_1_* 28 h started to be significant at early and late Phase I, respectively, and the effect sizes reduced at Phase II. The *a_1_* and *a_2_* estimates were all negative (Fig. 6c) and explained most of the total variances at 6 to 96 h, while *i_a1a2_* was significant only at 6, 10, 12, or 96 h (Supplementary Table 4). These data suggest that the 2 pigmentation genes are both involved in the regulation of imbibition speed for dormancy-released seeds, with the inhibitory effect on water uptake occurring earlier for *Pb* than for *Rc*.

## Discussion

The *Rc* and *Pb* genes were discovered as single Mendelian factors for the red and purple pericarp color traits, respectively, and both encode the bHLH family TFs for the PA or AC biosynthesis in rice. This research examined the trait development and quantified pleiotropic effects of the 2 loci on seed flavonoids, primary dormancy, and germination velocity by assembling the *Rc/rc* and *Pb/pb* alleles into the same genetic background. Four development patterns of the pigment traits were identified in the digenic system, based on differences in the temporal distribution of pericarp colors from 5 to 40 DPA and in the pigment subcellular compartmentation in the lignified lower epidermal area (Figs. 1b and 2b). Two subgroups of 5 types of flavonoid metabolites, including AC and PA, were detected in both developing and developed caryopses (Table 1). In addition to *Rc* (Gu et al. 2011), *Pb* also has a pleiotropic effect on seed dormancy in 2 experiments (Fig. 4). Both the *Rc* and *Pb* loci were associated with seed imbibition and germination velocities when the dormancy was released (Figs. 5 and 6). Epistatic interactions between the 2 loci contributed to variations for the trait development pattern, AC (not PC) content, dormancy degree, and germination velocity. Development times and their interactions with the *Rc* or *Pb* genotypes influenced the flavonoid accumulation in the coat tissue. Seed adaptive traits are critical for functional ecology (Saatkamp et al. 2019). The listed lines of information could help explain the similarity and difference in functionality between seed pigment traits.

### About the adaptive difference between seed pigment traits

An adaptive difference between genotypes in a population arises when some phenotypes are better suited to local environments than others. For example, it was red, not purple, pericarp-colored genotypes that prevail in wild and weedy rice (Oka 1988; Sweeney et al. 2006; Delouche et al. 2007; Gross et al. 2010). This research provided 2 lines of information that could help explain the adaptive difference between the red and purple pigment traits. The first is the 2 subgroups of flavonoids produced in the *Rc*- or *Pb-*pigment systems. The PA flavon-3-ols (dimers) differ from the AC monomers in molecular structure and water-solubility. The dimers may provide a better coating effect than the water-soluble monomers, as suggested by the pigment compartment patterns at 15 DPA (Fig. 2b). And the second is the difference in the size of genic effect on seed primary dormancy between the *Rc* and *Pb* loci (Table 2). The different effects suggest that the red pericarp-colored seeds have stronger dormancy than the purple ones. However, the 2 lines of information cannot explain why the genotypes with both red and purple pigments were not reported for the non-domesticated rice. Thus, there must be additional mechanisms contributing to the adaptive difference between the 2 pigment traits, such as soil seedbank longevity and seed aging tolerance (Pipatpongpinyo et al. 2020; Prasad et al. 2023).

### About the difference in genotypic frequency between the red- and purple-colored specialty varieties

The spatiality varieties were/are selected because of nutritional values and health benefits of pigmented caryopses (Mbanjo et al. 2020). However, the frequency distribution in the collection of 696 pigmented accessions is dramatically biased toward the red pericarp-colored genotypes (81.47%) and the genotypes were confirmed with the presence of the *Rc* allele (Mbanjo et al. 2019). The *Rc* system produced more catechins (10.2 mg/g) than PAs (3.6 mg/g), suggesting that the red pigments are not the only reason for the biased selection. In addition, the content of the total flavonoids in mature caryopses was much greater for the flavan-3-ols (14 mg/m) in the red- than for the anthocyanins (3.3 mg/g) in the purple-pigmented genotype. Research is needed to identify differences in the nutritional and/or health advantages between the 2 subgroups of flavonoids and between the flavan-3-ols. Breeders may combine the *Rc* and *Pb* alleles in 1 variety to produce the 2 subgroups of dietary flavonoids, with the contents like those in the genotypes containing only 1 of the alleles.

## Supplementary Material

jkaf158_Supplementary_Data

## Data Availability

The authors affirm that all data necessary for confirming the conclusions of the article are present within the article, figures, tables, and Supplementary Material. Supplementary Material includes the list of genes identified by genetic analysis for red or purple pericarp traits in rice (Supplementary Table 1), all the primers used in this research (Supplementary Table 2), summary of genetic component effects of the *Rc* and *Pb* loci on flavonoid content (Supplementary Table 3), summary of statistics for additive and epistatic effects of the *Rc* and *Pb* loci for water content of germinating seeds content (Supplementary Table 4), morphological and genotypic differences among the 4 ILs (Supplementary Fig. 1), and allelic variations for genomic DNA sequence at *OsB1* and *OsB2* (Supplementary Fig. 2). Supplemental material available at G3 online.
