# Correction to: Pleiotropic effects of red and purple pericarp genes on seed coating patterns, flavonoids, dormancy, and germination in rice

**DOI:** 10.1093/g3journal/jkaf210

**Published:** 2025-09-12

This is a correction to: Wenwu Tang, Min Guo, Yue Zhu, Rupak Chakraborty, Bhupinder S Batth, Kamal Bhattarai, Guiquan Zhang, De-Yu Xie, Xing-You Gu, Pleiotropic effects of red and purple pericarp genes on seed coating patterns, flavonoids, dormancy, and germination in rice, *G3 Genes|Genomes|Genetics*, 2025; https://doi.org/10.1093/g3journal/jkaf158

The following changes have been made to the originally published manuscript. The affiliations of co-authors Yue Zhu and De-Yu Xie are emended to read: “Department of Plant and Microbial Biology, North Carolina State University, NC 27695, United States” instead of: “Biology Department, North Carolina State University, Raleigh, NC 27695, United States”.

