# Supplementary figures and images for: Pleiotropic effects of red and purple pericarp genes on seed coating patterns, flavonoids, dormancy, and germination in rice
Source: G3 (Bethesda). 2025 Jul 18;15(9):jkaf158. doi: 10.1093/g3journal/jkaf158 (PMC12405871; doi:10.1093/g3journal/jkaf158)

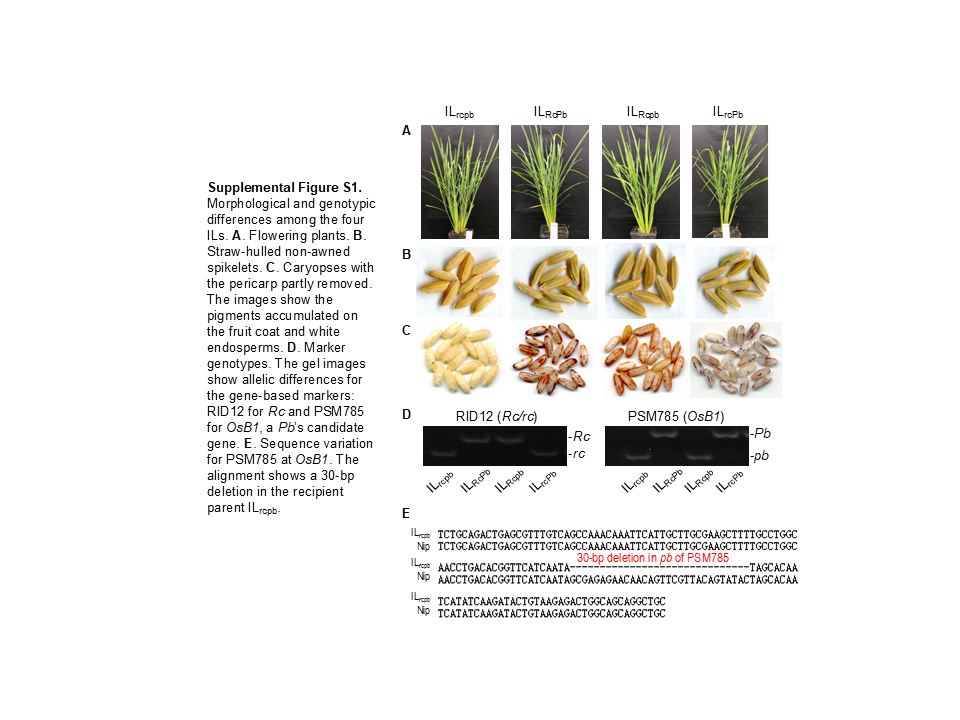

Supplement: jkaf158_Supplementary_Data [file jkaf158_supplementary_data.zip › Figure_S1_G3-2025-406002.tif]

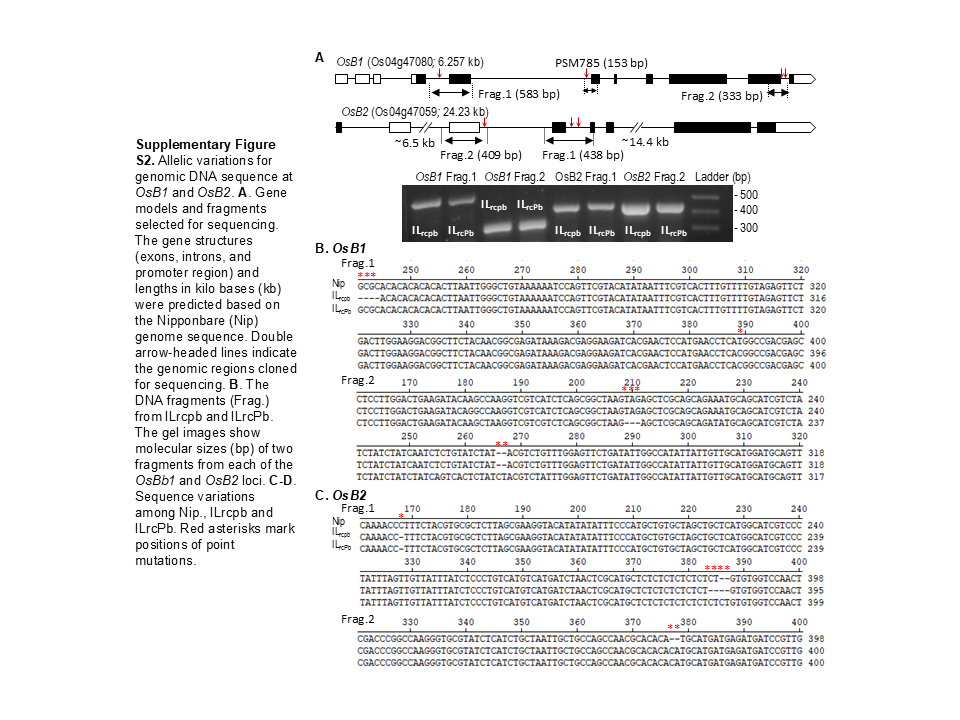

Supplement: jkaf158_Supplementary_Data [file jkaf158_supplementary_data.zip › Figure_S2_G3-2025-406002.tif]
